# Prevalence of Oral Lesions in Hospitalized Patients with Infectious Diseases in Northern Brazil

**DOI:** 10.1155/2014/586075

**Published:** 2014-01-15

**Authors:** Karina Gemaque, Gustavo Giacomelli Nascimento, José Luiz Cintra Junqueira, Vera Cavalcanti de Araújo, Cristiane Furuse

**Affiliations:** ^1^São Leopoldo Mandic Institute and Research Center, Rua José Rocha Junqueira 13, 13045-755 Campinas, SP, Brazil; ^2^Federal University of Pelotas, Pelotas School of Dentistry, Rua Gonçalves Chaves 457, 96015-560 Pelotas, RS, Brazil; ^3^Araçatuba School of Dentistry, Universidade Estadual Paulista (UNESP), Rua José Bonifácio 1193, 16015-050 Araçatuba, SP, Brazil

## Abstract

The aim of this study was to assess the prevalence of oral lesions in infectious-contagious diseases patients being treated in the University Hospital of the Federal University of Pará, northern Brazil. One hundred seven patients with infectious diseases were clinically investigated for oral lesions at the University Hospital of Pará, northern Brazil. From total sample, most patients were men (65.7%) with a mean age of 45.4 years. About prevalence of systemic diseases, tuberculosis was the most frequent illness, followed by AIDS, hepatitis types B and C, leishmaniasis, and meningitis. Analyzing oral manifestations, periodontal diseases and candidiasis were the most prevalent diseases in both genders, followed by recurrent aphthous ulcers, saburral tongue, simplex herpes, and squamous cell carcinoma. Of all 107 patients, only 10 males and 6 females did not present any oral manifestation. There was no statistical difference between genders with any systemic condition (*P* > 0.05). The great prevalence of oral manifestations in hospitalized patients with systemic disorder emphasizes the need of integral dental care in this context, aiming at a multidisciplinary approach of patients. Therefore, presence of some oral conditions, such as candidiasis, should be an alert to different systemic conditions, once in assistance with physicians; dentists can influence the early diagnosis and treatment.

## 1. Introduction

In the world, high population density, inadequate water supplies, and inadequate sewage disposal systems aided in the spread of infectious diseases and contributed to high urban mortality levels for most of the 19th century [1]. The “theory of epidemiologic transition” attributes these trends to the transition from an “age of pestilence and famine” in which the mortality pattern was dominated by high rates of infectious disease deaths especially in the young to the current “age of degenerative and man-made diseases” in which mortality from chronic diseases predominates. Until recently, it was assumed that the epidemiologic transition had brought about a permanent reduction in infectious disease mortality worldwide. However, the emergence or reemergence in the 1980s of such diseases as the acquired immunodeficiency syndrome (AIDS) and tuberculosis demonstrated that gains against infectious diseases cannot be taken for granted [2].

The tissues of the oral cavity frequently reflect the condition of a person's general health and often may indicate the presence of a systemic disease, since many lesions or diseases occur primarily within the oral cavity [3]. These lesions may be useful adjuncts to these clinical diagnoses and occasionally can be the presenting sign or symptom of a specific systemic disease, such as an infection by the HIV virus [4]. Notably, presence of some infectious diseases in mouth can still reveal patient's condition or a therapeutic response, being an adjunct to clinical conduct and prognostic. Dentists can play a key part in the diagnosis and management of patients and have an exceptional opportunity to become familiar with and to interpret changes in oral tissues. In cooperation with physicians, the dentists can often influence the early diagnosis and treatment. A comprehensive plan for management of the nonspecific conditions and the side effects of the specific diseases is available and can be used by dentists in the supportive care of the mouth [5].

In Brazil, available data about health services are not sufficient for an adequate assessment of trends at a national level and can be underestimated for social and political issues. Moreover, for being a country with continental dimensions divided into five geographical regions with differing demographic, economic, social, cultural, and health conditions and widespread internal inequalities, distribution of infectious diseases is heterogeneous [6]. The north region, which contains most of the Amazon rainforest, has the country's lowest population density (3.9 people per km²) and is the second poorest region; besides, there is a lack of studies and information about this region [6]. Consequently, this study aims to assess the prevalence and alterations in oral cavity of infectious-contagious diseases patients being treated in the University Hospital of the Federal University of Pará, northern Brazil.

## 2. Material and Methods

The study was approved by the Local Ethics Committee (Hospital Universitário João de Barros Barreto, Universidade Federal do Pará, Protocol no. 0943/08). This was a cross-sectional study performed in the city of Belém, northern Brazil, between June and August 2008. Hospitalized patients (*n* = 107) from the Department of Infectious-Contagious Diseases, the University Hospital of the Federal University of Pará, Brazil, constituted the population for the study. Patients signed a consent form to participate in the study. Informed consent forms for patients below legal age were obtained from their parents or legal representatives.

Inclusion criteria included patients with diagnosis of infectious-contagious diseases, education, and ability to understand and answer the questionnaire. Exclusion criteria included systemic diseases such as nutritional deficiency; cardiovascular, respiratory, metabolic, and endocrinal disorders; and history of an intervention or condition that could affect the oral mucosa (e.g., history of radiotherapy).

After obtaining the written informed consent of subjects, a questionnaire was administered to gather demographic details and medical history. Head, neck, and oral examinations were conducted by one dentist, calibrated by senior oral physicians using a standard set of color slides. Periodontal disease was measured in three sites (mesiobuccal, buccal, and distolingual sites) of all presented teeth, using William's probe. Two measures were recorded: gingival recession and probing depth. The combined attachment loss (CAL) for each site was calculated by summing gingival recession and probing depth. The diagnostic of periodontal disease was defined by the association of gingival bleeding and clinical attachment loss (CAL) ≥ 4 mm in two or more sites [7]. Biopsies and exfoliative cytology were performed according to diagnostic needs.

Data were submitted to descriptive analyses, and the Chi-square or Fisher's exact test was also performed in order to test the association between the independent variables and gender. Analyses were carried out with Stata 10.0 (StataCorp, College Station, TX, USA) software package and a level of significance of *P* ≤ 0.05 was considered.

## 3. Results

Infectious-contagious diseases patients (*n* = 107) hospitalized in the University Hospital of the Federal University of Pará, Brazil, constituted the population for the study. From the total of sample, 68 (63.5%) patients were male and 39 (37.5%) were female. About age, mean age was 45.4 years (SD 7.89) ranging from 13 to 97 years old.

About prevalence of infectious-contagious diseases, tuberculosis was the most prevalent illness (40.2%), followed by AIDS (32.7%), hepatitis types B and C (13.1%), leishmaniasis (9.3%), and meningitis (4.7%) (Table 1). Analyzing oral manifestations, 84.3% of all patients presented at least one oral sign. However, it is important to note that patients with tuberculosis and leishmaniasis did not present typical lesions from these specific illnesses in the mouth. Periodontal diseases (57.5%) and candidiasis (22.8%) were the most common lesions observed in both gender, followed by recurrent aphthous ulcers (12.6%), coated tongue (5.5%), simplex herpes (0.8%), and squamous cell carcinoma (0.8%). Of all 107 patients, only 10 male (9.8%) and 6 female (5.9%) patients did not present any oral manifestation. Distribution of lesions/alterations according to gender and systemic disorder is presented in Table 2. Statistical analysis did not reveal differences between gender with any systemic disease (*P* > 0.05).

Analyzing specific manifestations of candidiasis in different clinical presentations, it was possible to note that, in candidiasis, pseudomembranous type was present in 10 male and 9 female patients, resulting in 65.5% of all candidiasis cases; erythematous type was present in 4 male and 5 female patients (31.0%); angular cheilitis was present in only one female patient (3.4%).

In relation to anatomic location of findings, periodontal tissue was the most affected site, once gingivitis and periodontitis were the most prevalent diseases. Other than periodontal tissue, dorsal tongue was the second most affected site (31.6%), followed by lateral border of tongue (28%), hard palate (15.8%), buccal mucosa (14.0%), apex of tongue (8.8%), lips mucosa (5.3%), and soft palate (1.7%). It is important to highlight that number of lesions and sites is greater than number of patients, since many subjects presented more than one lesion in mouth.

## 4. Discussion

To the best of our knowledge, this is the first study conducted in a tertiary hospital analyzing the prevalence of oral manifestations in infectious diseases. Studies have been describing oral manifestations in systemic alterations or in infectious diseases separately, such as HIV infection or tuberculosis. Our study also presents data from typical diseases from developing countries, like leishmaniasis and hepatitis. It is important to highlight that data could be representative of the north region of Brazil, once the hospital analyzed is a reference for all northern regions.

Many reports have demonstrated how systemic health and oral health are linked. Oral examination can reveal signs and symptoms of immunologic diseases, endocrinopathies, hematologic conditions, systemic infections (virus, bacterial, and yeast infection), and nutritional disorders [8]. In addition, several studies have reported associations between periodontal disease and diabetes mellitus, heart disease, stroke, and adverse pregnancy outcomes [9]. Some oral changes are nonspecific, whereas others directly lead to the diagnosis of a particular disorder [10]. Some conditions are not even diseases, like coated tongue, but they can collaborate with a pathologic process.

In our study, infectious and nontraumatic disease, developmental disease, and neoplasia were the most frequent oral lesions. Periodontal disease, especially severe and moderate types, and candidiasis, pseudomembranous, erythematous, and angular cheilitis types, in this sequence, were the most common alterations found, followed by recurrent aphthous ulcers, coated tongue, simplex herpes, and squamous cell carcinoma. Even with the great prevalence of periodontal diseases, this specific condition could not be related to any of the infectious diseases, once patients presented a poor oral health, reinforced by the lack of oral health care in hospitals. Moreover, candidiasis has been considered as one of most prevalent lesions associated with systemic disorders, with ranges varying from 5 to 92% [11, 12]. This fact could be related to many factors such as the presence of *Candida* in oral cavity in healthy conditions [11] and the opportunistic nature of this yeast, being associated with severe immunosuppression, in special, the pseudomembranous type [12].

Our findings are corroborated by different reports evaluating specially HIV patients [13, 14]. In our sample, more than 90% of HIV patients presented oral manifestation. It is important to note that authors have suggested that different alterations are observed in developed and developing countries [15, 16]. Findings from the developing world are consistent with those from the developed countries in that oral candidiasis is generally the most frequently seen oral HIV lesion. Moreover, the prevalence of HIV-associated gingival/periodontal disease appears to be higher in developing countries. In the developing world, this may be associated with inadequate oral hygiene regimes and nutritional deficiencies [15]. Lesions resembling aphthous ulcers have been reported in patients at more or less similar prevalence in both the developing and developed world [17, 18]. The specific cause of these aphthous ulcerations in HIV patients remains unclear, but investigators have proposed a number of possible contributing factors, including overstimulation of tumor necrosis factor [19], immune cross-reactivity to infectious agents, nutritional deficiencies, stress, and hormonal imbalance [20]. In our sample, only 6 patients presented oral ulcers (5.6%) according to findings from Ranganathan and Hemalatha [14]. A reasonable explanation for the absence of oral hairy leukoplasia and Kaposi's sarcoma could be the use of antiretroviral therapy. The few studies conducted in the era of this therapy reported a decline in the prevalence of these lesions [21]. A low prevalence of simplex herpes was observed in our study (0.7%), and similar findings were detected in India (0.9%) and in Nigeria (2.5%). Use of antiretroviral therapy could also influence these rates since its presence occurs in association with low CD4+ lymphocytes number [22]. Other lesions such as squamous cell carcinoma have been reported [15]. Its presence could be ascribed to sociocultural habits, but its role in the development of squamous cell carcinoma in HIV-positive individuals is poorly understood and warrants further investigation.

An important condition verified was the association of oral lesions (more than one lesion in same patient). It was observed in 15 individuals from our sample, totalizing 26.3% subjects, specially affecting male. Most common associations were candidiasis with periodontal disease and with recurrent aphthous ulcers. Sulka et al. [23] and Ranganathan and Hemalatha [14] also have shown an association of lesions, suggesting that prognosis is disfavorable, requiring a combined therapeutic in most of times.

Patients with tuberculosis did not present any oral manifestations. According to previous reports, oral manifestations are uncommon, observed only in 0.05 to 5% of patients with tuberculosis. Most of these cases represent lesions secondary to pulmonary disease, once the primary form is uncommon in the oral cavity. Even being a rare condition, oral tuberculosis ulcers are more often reported in patients from the developing world [24, 25] and most of cases also represent lesions secondary to pulmonary tuberculosis [26].

Patients with hepatitis B and C presented periodontal disease and candidiasis as the most common alterations. Reports of oral manifestations in hepatitis B and C are not frequent, especially for HBV infection. Xerostomia, Sjögren's syndrome, sialoadenitis, and oral lichen planus have been the main conditions associated with HCV infection in the literature [27]. A reason for the absence of lichen planus cases in our sample could be the reduced number of patients with this infection.

Among the 10 patients with leishmaniasis, only one presented the nasal mucosa leishmaniasis with nasal septum resorption. Oral lesions usually appear as ulceration in the hard or soft palate [28, 29]. In this study, no typical intraoral manifestation of leishmaniasis was observed, although subjects had unspecific lesions such as candidiasis and periodontal disease. Cases of meningitis were the rarest of our sample (5; 4.7%), and only two of them presented oral manifestations: periodontal disease and candidiasis. It is important to emphasize that those lesions are not specific from meningitis, once they are also prevalent in other infectious diseases [13, 15].

Since many systemic disorders have oral manifestations, this component may precede the systemic presentation of a particular disease. Early diagnosis and management can often diminish the morbidity associated with a systemic disease. Careful examination of the oral cavity is a necessary component of the diagnostic workup for any patient.

## 5. Conclusion

Tuberculosis and HIV infection were the most prevalent infectious diseases among the studied population. Even though typical alterations in oral cavity were not observed, a high rate of unspecific manifestations was also found, especially candidiasis and periodontal disease of severe and aggressive types, suggesting an immunological suppression of those patients. The great prevalence of oral manifestations in hospitalized patients with systemic disorder emphasizes the need of integral dental care in a medical context, aiming for a multidisciplinary approach of these subjects. Therefore, presence of some oral conditions, such as candidiasis, should be an alert to different systemic conditions, once in assistance with physicians; dentists can influence the early diagnosis and treatment.

## Figures and Tables

**Table 1:** Distribution of systemic disorder according to gender. Pará, Brazil (*n* = 107).

	Women	Men	Total
	*n* (%)	*n* (%)	*n* (%)
HIV infection/AIDS	11 (10.3)	24 (22.4)	35 (32.7)
Meningitis	3 (2.8)	2 (1.9)	5 (4.7)
Leishmaniasis	2 (1.9)	8 (7.5)	10 (9.4)
Tuberculosis	18 (16.8)	25 (23.4)	43 (40.2)
Hepatitis	5 (4.7)	9 (8.4)	14 (13.1)

Total	39 (36.5)	68 (63.5)	107 (100.0)

**Table 2:** Distribution of oral lesions/alterations according to systemic disorder and gender. Pará, Brazil (*n* = 107).

	Periodontal disease		Candidiasis		Recurrent aphthous ulcers		Coated tongue		Simplex herpes		Squamous cell carcinoma	
	Women	Men	Women	Men	Women	Men	Women	Men	Women	Men	Women	Men
HIV infection/AIDS	9 (6.6)	19 (13.9)	5 (3.6)	8 (5.8)	1 (0.7)	5 (3.6)	—	1 (0.7)	1 (0.7)	—	—	1 (0.7)
Meningitis	2 (1.4)	3 (2.2)	1 (0.7)	—	—	—	—	—	—	—	—	—
Leishmaniasis	1 (0.7)	5 (3.6)	—	1 (0.7)	1 (0.7)	3 (2.2)	—	—	—	—	—	—
Tuberculosis	10 (7.4)	19 (13.9)	9 (6.6)	6 (4.4)	4 (2.9)	1 (0.7)	—	4 (2.9)	—	—	—	—
Hepatitis	5 (3.6)	5 (3.6)	3 (2.2)	1 (0.7)		1 (0.7)	1 (0.7)	1 (0.7)	—	—	—	—

Total	27 (19.6)	51 (37.2)	18 (13.1)	16 (11.7)	6 (4.4)	10 (7.4)	1 (0.7)	6 (4.4)	1 (0.7)	—	—	1 (0.7)
